# Improvements in newborn care and newborn resuscitation following a quality improvement program at scale: results from a before and after study in Tanzania

**DOI:** 10.1186/s12884-014-0381-3

**Published:** 2014-11-19

**Authors:** Christina Lulu Makene, Marya Plotkin, Sheena Currie, Dunstan Bishanga, Patience Ugwi, Henry Louis, Kiholeth Winani, Brett D Nelson

**Affiliations:** Jhpiego Tanzania, PO Box 9170, Dar es Salaam, Tanzania; Jhpiego Baltimore, Baltimore, MD USA; Departments of Pediatrics and Emergency Medicine, Massachusetts General Hospital, Boston, MA USA; Tanzania Ministry of Health and Social Welfare, Dar es Salaam, Tanzania; Harvard Medical School, Boston, MA USA

**Keywords:** Essential newborn care, Newborn resuscitation, Newborn health, Quality of care, Tanzania

## Abstract

**Background:**

Every year, more than a million of the world’s newborns die on their first day of life; as many as two-thirds of these deaths could be saved with essential care at birth and the early newborn period. Simple interventions to improve the quality of essential newborn care in health facilities – for example, improving steps to help newborns breathe at birth – have demonstrated up to 47% reduction in newborn mortality in health facilities in Tanzania. We conducted an evaluation of the effects of a large-scale maternal-newborn quality improvement intervention in Tanzania that assessed the quality of provision of essential newborn care and newborn resuscitation.

**Methods:**

Cross-sectional health facility surveys were conducted pre-intervention (2010) and post intervention (2012) in 52 health facilities in the program implementation area. Essential newborn care provided by health care providers immediately following birth was observed for 489 newborns in 2010 and 560 in 2012; actual management of newborns with trouble breathing were observed in 2010 (n = 18) and 2012 (n = 40). Assessments of health worker knowledge were conducted with case studies (2010, n = 206; 2012, n = 217) and a simulated resuscitation using a newborn mannequin (2010, n = 299; 2012, n = 213). Facility audits assessed facility readiness for essential newborn care.

**Results:**

Index scores for quality of observed essential newborn care showed significant overall improvement following the quality-of-care intervention, from 39% to 73% (*p* <0.0001). Health worker knowledge using a case study significantly improved as well, from 23% to 41% (*p* <0.0001) but skills in resuscitation using a newborn mannequin were persistently low. Availability of essential newborn care supplies, which was high at baseline in the regional hospitals, improved at the lower-level health facilities.

**Conclusions:**

Within two years, the quality improvement program was successful in raising the quality of essential newborn care services in the program facilities. Some gaps in newborn care were persistent, notably practical skills in newborn resuscitation. Continued investment in life-saving improvements to newborn care through the health services is a priority for reduction of newborn mortality in Tanzania.

## Background

Every year, more than a million of the world’s newborns die on their first day of life [1]. Although significant progress has been made over the past two decades in child survival, less progress is evident in reducing newborn mortality, especially in sub-Saharan Africa. Neonatal deaths account for 43% of under-five child deaths globally and account for 40% of under-five mortality in Tanzania [2,3]. Two-thirds of all newborn mortality (deaths in the first 28 days of life) occurs in 12 countries, six of which are in sub-Saharan Africa, and this includes Tanzania [4]. In Tanzania, infant mortality (deaths in the first year of life) has seen dramatic reduction in the past 13 years, falling from 71/1,000 to 51/1,000 live births, but neonatal mortality has remained more constant in the same period, from 30/1000 to 26/1,000 [5]. Helping newborns survive their first day is a priority in the work of the Tanzanian Ministry of Health and Social Welfare (MOHSW) and of other key partners working to accelerate progress toward Millennium Development Goal (MDG) 4 for child survival [6].

Most causes of newborn mortality are preventable or treatable. Cost-effective, evidence-based interventions for prevention and treatment of the major causes of newborn mortality – prematurity, birth asphyxia, and infections – are well established [7–10] and as well, studies have shown that use of quality improvement initiatives can be effective in applying these interventions to cause at least a moderate reduction in newborn mortality [11]. However, massive gaps remain in the quality and coverage of these interventions both through the health care infrastructure and at community level. Good quality care during labor and birth and following childbirth is particularly important, as this is the period in which most lives are saved. Key essential newborn care interventions to reduce morbidity and mortality include immediate drying and warming including skin-to-skin care, clean cord care, resuscitation for newborns with birth asphyxia, and early and exclusive breastfeeding for all newborns [12].

Birth asphyxia, or intrapartum-related hypoxic events, is a major contributor to newborn mortality, causing an estimated 23% of newborn deaths globally [13]. As many as two-thirds of the 3 – 6% of newborns who are born with birth asphyxia could be saved with interventions at birth, including clearing of the airway, tactile stimulation, and assisted ventilation with a bag-and-mask device. Assisting newborns with ventilation can reduce mortality among neonates by approximately 30% [14]. A 2013 study by Msemo et al. in nine Tanzanian hospitals showed that provision of newborn resuscitation – specifically, by skilled birth attendants trained in Helping Babies Breathe (HBB) – reduced neonatal mortality by 47% [15]. HBB is an evidence-based educational programme to teach newborn resuscitation techniques in resource-limited areas [16].

According to van den Broek, the provision of quality care should be the central focus of efforts to achieve MDG targets for maternal and newborn health [17]. The Tanzanian MOHSW has endorsed various quality improvement initiatives, including, in 2009, the 5S Approach, which focuses on facility-based infrastructure and attitude improvements, as well as quality improvement for maternal and newborn care using nationally approved basic emergency obstetric and newborn care (BEmONC) performance standards [18]. Our current study conducted an evaluation of the quality of newborn care associated with a USAID-funded program to improve maternal and newborn health services in Tanzania, run collaboratively between a university-affiliated non-governmental organization and the MOHSW of Tanzania. Key programmatic approaches included training for health care providers (nurses, midwives, clinical officers, and assistant medical officers) in BEmONC and routine delivery care, provision of essential equipment (e.g., bag-and-mask device, suction), supportive supervision of BEmONC in health care facilities, a quality improvement approach in facilities based on national BEmONC standards, and improvements to national health information systems for maternal and newborn health. BEmONC training is conducted using a nationally approved learning package: the training is a 13-day competency-based in-service training, which utilizes didactic approaches, simulation, actual clinical practice and proficiency assessment. (The HBB-specific newborn resuscitation curriculum was not yet available at the time of this program but is since being implemented through a training program which is being rolled out to health care providers nationally). While prematurity is notably a leading cause of death of newborns in Tanzania, the MAISHA program focused more heavily on the management of birth asphyxia, as well as supporting care of low birth weight or pre-term babies through kangaroo mother care.

The program trained and provided quality improvement support to more than 1,593 providers and supervisors from 251 facilities nationwide (including mainland Tanzania and Zanzibar) from 2009 to 2013. The quality improvement process utilized the Standards Based Management and Recognition (SBM-R) approach, in which facility-based quality improvement teams are brought together, trained, and subsequently conduct internal quality assessments utilizing national BEmONC standards. Facilities are externally assessed annually or upon request, and facilities reaching an 80% score are recognized by the MOHSW.

In the 52 facilities assessed, the program trained a total of 243 service providers in newborn care (average 3 providers in lower level facilities, 11 providers in regional hospitals). Not all providers of maternity services in these facilities, and thus providers assessed in the study, had been trained by the program. However, onsite coaching and quality improvement initiatives involved all providers working in maternity at time of coaching.

The program aimed at decongesting hospitals by improving quality of care at lower level health facilities, which are closer to communities. Quality MNH services at dispensary and health center would encourage women to seek services from those sites, and reduce referral of to overcrowded hospitals. The program thus worked to improve quality of care in regional hospitals, as training centers for the region, and lower level health facilities (health centers and dispensaries).

This article reports on the changes in quality of newborn care before and two years following the implementation of this program. The study was conducted in facilities in 12 of Tanzania’s 30 regions, which were the regions reached in Year 1 and 2 rollout of the program.

Internationally, there are relatively few widely used, standardized assessments of the quality of maternal and newborn health care [19,20]. The quality-of-care surveys that do exist largely focus on facility readiness, based on facility audits of human resources, availability of equipment and commodities, and self-reported practices of health care workers [17,21]. This study is among few that are based on direct observations of maternal and newborn care and, as such, provides important new insight into the quality of newborn health care services in a developing country.

## Methods

### Overview

From July – August 2010, the program conducted a cross-sectional health facility survey to gather baseline information on the quality of maternal and newborn care in facilities targeted for support in 12 regions of Tanzania included in the first year of program rollout (Tanga, Mtwara, Lindi, Arusha, Kilimanjaro, Morogoro, Manyara, Tabora, Pwani, Kigoma, Ruvuma, Iringa). In November – December 2012, following approximately two years of intervention in these facilities, the survey was conducted again to document changes in the quality of care. The study sample was based on uncomplicated deliveries, but management of complications, including management of newborns needing resuscitation, was also recorded using a standardized checklist.

Related to quality of essential newborn care and resuscitation skills of health care providers, this study assessed the following areas in the following ways:

1. Provider compliance with global clinical standards in essential newborn care: the care provided to women giving birth and their newborns in the immediate postpartum period and up to one hour following birth was observed and documented by standardized observers using an observation checklist, based on WHO best-practice guidelines for maternal and newborn care [22].
2. Health worker knowledge and newborn resuscitation skills during simulated case scenarios using a mannequin: Provider knowledge was assessed by presenting a case study to the provider and then administering a questionnaire with a series of knowledge questions on maternal and newborn care. Skills were assessed by the observer using a standardized checklist to evaluate the provider conducting a simulated, structured resuscitation on a newborn mannequin (NeoNatalie™) [23]. The knowledge assessment questionnaire and observations on the mannequin were conducted together for selected health care providers.
3. Case management of actual resuscitations of newborns: case management of newborns receiving resuscitation was observed by the standardized observers using a structured observation checklist embedded into the observation tool. Observers, who were trainers in maternal and newborn health, intervened in cases in which the care being provided was putting the life of the mother or newborn at risk.
4. Availability of equipment and supplies for newborn resuscitation and essential newborn care: availability of supplies, equipment, and infrastructure for maternal and newborn services was assessed using a facility audit tool, which was applied in the labor, delivery, antepartum and postpartum areas and in the facility’s pharmacy. The study assessed availability of supplies and equipment on the day of data collection. The key supplies assessed for newborn care were: sterile scissors or blade to cut cord; functional suction equipment; resuscitation table; functioning bag and mask.

### Sampling

This study was designed to generate point estimates of the quality of routine maternal and newborn practices during intrapartum and immediate postpartum care up to one hour after birth. In both 2010 and 2012, the targeted number of deliveries to be observed was 449. This was based on known performance of a key service for normal delivery (provision of active management of third stage of labor, baseline of 7%), a detection of change of up to 50% with 80% power and 95% precision. A separate sampling process was conducted for Zanzibar, and these data are not presented in this paper. Women admitted for emergency cesarean section were not observed, nor were their newborns.

The program focused on improving quality of care in regional hospitals, health centers, and dispensaries and this is reflected in the sample of health facilities in the study. District hospitals were not included in the program and, thus, not included in the facilities sampled into the study. In 2010, all health care facilities that started receiving the intervention within the first two years of the maternal and newborn health program were listed, and facilities with an average of at least one delivery per day (365 deliveries per year) were considered to be ‘high volume’ and were included in the study, resulting in 52 facilities. Very few dispensaries met the criteria of at least one delivery per day, and, therefore, for the purpose of analysis, these dispensaries were included with the health centers. In 2012, all of the baseline facilities were revisited, with the exception of two health centers that had not received the intervention as planned due to staffing limitations, resulting in 50 health facilities. A quota on the number of deliveries to be observed at each facility during the 2- to 4-day data collection period was calculated based upon the expected number of deliveries and the number needed in the study for statistical significance.

For the knowledge and skills assessments using the newborn mannequin, a convenience sample was taken of six health care providers working in the maternity ward per hospital and four per lower-level health facility. These were selected among health care providers on-shift during the data collection period. While providers’ knowledge scores were not linked to observations of care provided, all of the providers who had their knowledge assessed were also observed conducting deliveries and providing newborn care.

### Data collection

Data collectors were national BEmONC trainers who had undergone a one-week training on study methodology, research ethics, and orientation to and practice using the study tools and mobile devices, including a practicum session in a health facility. In order to reduce bias, data collectors were assigned to conduct observations in regions where they had never previously trained health care providers. Fourteen of the twenty data collectors in 2012 were the same as those in 2010. Inter-rater reliability was assessed during the training to improve standardization among data collectors.

One data collector observed not more than three births simultaneously. Observers entered data on delivery and newborn care directly into tablets or smart phones (Samsung Galaxy Tablets with Mobile Data Studio software [24]) that were pre-populated with the data collection forms. The data entry applications controlled question flow, data skips along with range, consistency and some data quality checks. Every evening the supervisors would transmit data to an online server, which allowed online monitoring. Upon receiving data on the server, other quality checks were performed before porting the cleaned data over to a SQL Server database and a password-protected web portal for analyzing and displaying the data in web tables and field work maps.

### Weighting of the data

The number of cases observed in each facility was proportional to the client volume. By observing the same period of time in each facility, the sample was designed to be self-weighted according to service volume; however, corrective weights had to be applied so that the sample matched the average service volume from the previous year. The corrective weight applied to each facility’s birth data has the value of the ratio of the number of births seen in that facility divided by the number of births expected in that facility from previous year’s maternity utilization data, which had been used to calculate the original sample. The corrective weight was applied so that the final sample was weighted according to the maternity utilization data.

### Analysis

Descriptive statistics, including means and frequencies, were prepared using Microsoft Excel 2010 and IBM SPSS Statistics 20. Case studies of newborn resuscitation were analyzed qualitatively, using tabulation of key events and reviewing case notes. Standard quantitative analyses were performed using Stata Statistical Software 11.0 (StataCorp, 2009) and Microsoft Excel 2011 (Redmond, WA, USA). These analyses included chi-square tests to compare quality-of-care indicators for newborn resuscitation and immediate newborn care between the years 2010 and 2012. An index score for the overall quality of essential newborn care was developed based upon four indicators: immediately drying the newborn, placing the newborn on the mother’s abdomen skin-to-skin, cutting the umbilical cord with a clean blade, and initiating breastfeeding within one hour of birth. Only observations in which all four steps were correctly performed received a point for this index score. Missing data were automatically assumed to have a score of 0. A p-value of 0.05 was considered statistically significant in all analyses.

### Ethics/institutional review

Permission to observe care was obtained from the facility managers, and health care providers and mothers observed provided verbal consent. Ethical clearance was obtained from by the Ethical Review Board of the Tanzania National Institute of Medical Research (Reference No. NIMR/HQ/R.8C/Vol II/164) and the Johns Hopkins Bloomberg School of Public Health Institutional Review Board (IRB No. 00002549).

## Results

### Essential newborn care

In the 52 facilities sampled into the study (Table 1), 489 newborns were observed in 2010 during their immediate postpartum period and 560 newborns were observed in 2012 (Table 2). In both years, at least one observation was conducted at every facility visited visited – the range of observations conducted was 216–294 in health centers/dispensaries and 195–344 in regional hospitals.

**Table 1:** **Number of facilities and facility types included in the quality of maternal and newborn health care survey, Tanzania, 2010–12**

	**Number (%) of facilities assessed in evaluation**	
	**2010**	**2012**
Total	52	50
**Region:**		
Tanga	6 (12)	6 (12)
Arusha	5 (10)	5 (10)
Lindi	3 (6)	3 (6)
Morogoro	5 (10)	5 (10)
Manyara	5 (10)	4 (8)
Iringa	4 (8)	4 (8)
Tabora	6 (12)	6 (12)
Kilimanjaro	5 (10)	5 (10)
Kigoma	3 (6)	3 (6)
Pwani	3 (6)	3 (6)
Mtwara	3 (6)	3 (6)
Ruvuma	4 (8)	4 (8)
**Facility type:**		
Regional hospital	12 (23)	12 (24)
Health centre/Dispensary	40 (77)	38 (76)

**Table 2:** **Observations and assessments in quality of care study, Tanzania, 2010–12**

	**Number of newborn observations**		**Number of resuscitations observed**		**Median number of health workers assessed per facility: skills**		**Median number of health workers assessed per facility: knowledge**	
	**2010**	**2012**	**2010**	**2012**	**2010**	**2012**	**2010**	**2012**
Total	419	504	37	40	299	213	206	217
**Region:**								
Tanga	52	41	6	0	20	24	22	24
Arusha	32	47	2	2	16	19	15	22
Lindi	16	20	2	1	13	12	13	12
Morogoro	23	34	8	1	25	18	25	19
Manyara	24	29	3	1	16	10	21	10
Iringa	68	29	5	4	24	11	20	13
Tabora	27	52	2	8	16	17	12	18
Kilimanjaro	36	70	3	1	18	40	22	40
Kigoma	4	41	1	8	13	21	16	21
Pwani	98	60	6	6	10	14	13	14
Mtwara	16	40	0	2	10	8	12	8
Ruvuma	27	41	4	6	19	15	15	16
**Facility type:**								
Hospital	195	344						
Health center/Dispensary	294	216						

Table 3 details the assessment of essential newborn care at the two time points. Overall achievement on the index score for newborn care went from 39% to 73%, representing an increase of 34 percentage points (<0.0001). High achievement in cord care and drying and wrapping of the newborn was seen at both baseline and follow-up. The areas that showed the most improvement were placing the newborn on the mother’s abdomen skin-to-skin immediately after birth (an increase of 35 percentage points, *p* <0.0001) and helping to initiate breastfeeding within one hour (42% increase, *p* <0.0001).

**Table 3:** **Immediate newborn care practices in the quality of maternal and newborn health care survey, 2010 and 2012**

	**Regional hospitals**				**Health centers and dispensaries**				**All facilities**			
	**2010 N = 168 (%)**	**2012 N = 317 (%)**	**Percent change**	**p-value**	**2010 N = 251 (%)**	**2012 N = 187 (%)**	**Percent change**	**p-value**	**2010 N = 419 (%)**	**2012 N = 504 (%)**	**Percent change**	**p-value**
Immediately places newborn on the mother's abdomen	72 (43)	241 (76)	33%	<0.0001	93 (37)	144 (77)	40%	<0.0001	165 (40)	385 (77)	37%	<0.0001
Immediately dries baby with towel	158 (94)	301 (95)	1%	0.8	210 (84)	181 (97)	13%	<0.0001	368 (88)	482 (95)	7%	0.0001
Discards wet towel and covers with dry towel	158 (94)	304 (96)	2%	0.4	213 (85)	181 (97)	12%	<0.0001	371 (89)	485 (96)	6%	<0.0001
Cuts cord with clean blade	168 (100)	317 (100)	0%	1	251 (100)	187 (100)	0%	1	419 (100)	504 (100)	0%	1
Helps initiate breastfeeding within one hour	67 (40)	263 (83)	43%	<0.0001	138 (55)	163 (87)	32%	<0.0001	205 (49)	401 (80)	31%	<0.0001
Ties or clamps cord when pulsations stop, or by 2–3 minutes after birth (not immediately after birth)	123 (73)	270 (85)	12%	0.001	183 (73)	155 (83)	10%	0.01	306 (73)	425 (84)	11%	<0.0001
Index score for overall quality of immediate newborn care*	49 (29)	235 (74)	45%	<0.0001	113 (45)	135 (72)	27%	<0.0001	162 (39)	370 (73)	34%	<0.0001

*The index score is based on four indicators: 1) immediately drying the newborn, 2) placing the newborn skin-to-skin, 3) cutting the umbilical cord cleanly, and 4) breastfeeding within one hour.

Universal adherence to cord cutting with a clean blade (e.g., sterile pair of scissors) was seen in both 2010 and 2012. Delayed cord clamping significantly increased over the two years, by 12% in regional hospitals (*p* = 0.0009) and by 10% in lower-level health facilities (*p* = 0.007). While end-line performance on overall essential newborn care steps was similar across levels of health care facility, lower-level health facilities were more likely than regional hospitals to promote immediate breastfeeding. This was statistically significant in 2010 (*p* = 0.0002) but not in 2012 (*p* = 0.08).

Although the WHO recommended practice of placing the newborn skin-to-skin on the mother’s abdomen immediately following delivery increased from baseline to endline, mothers and newborns were often subsequently separated in the hour following birth. While this study did not allow quantification of subsequent mother-newborn separation in the first hour, it was observed that newborns were often placed on the same bed alongside the mother, were taken to a separate room, or the mother left the delivery room to return to the antenatal area without the newborn, who was brought to her later.

### Newborn resuscitation skills and knowledge of essential newborn care

Health worker knowledge (assessed with the described knowledge assessment) improved significantly, from 23% in 2010 to 41% in 2012 (*p* <0.0001) (Table 4). Specifically, correct responses to questions on sepsis rose by approximately 15% (*p* <0.0001), and knowledge of equipment required for essential newborn care rose by 34% (p <0.0001) (data not shown).

**Table 4:** **Health worker knowledge and skills in newborn resuscitation, from simulated resuscitation with newborn mannequin and knowledge questions, 2010 and 2012**

	**2010**	**2012**	**p-value**
	**% of providers scoring correctly**	**% of providers scoring correctly**	
**Knowledge Assessment**	(N = 206)	(N = 217)	
Can state appropriate equipment and supplies for a newborn immediately after birth	15	49	<0.0001
Describes appropriate newborn care during the first hour of life	32	38	0.008
Recognizes signs of sepsis	21	36	<0.0001
**Total knowledge score**	**23**	**41**	**<0.0001**
**Skills Assessment using mannequin**	(N = 299)	(N = 213)	
***Positioning of the newborn mannequin for resuscitation:***			
Clears airway	75	80	0.3
Stimulates newborn	--	52	--
Places newborn on warm and clean surface	82	81	0.5
Places head in slightly extended position	60	77	0.001
All positioning tasks performed correctly	45	37	0.1
***Ventilation***			
Places correct size mask, covering chin, mouth, and nose	63	77	0.003
Checks seal by ventilating twice and observing chest rise	57	47	0.09
Ventilates at 30–50 breaths/minute	34	48	0.01
All ventilation tasks performed correctly	28	32	0.4
**Total skills score (all positioning and ventilation tasks performed correctly)**	**48**	**28**	**<0.0001**

Provider skills, assessed with structured clinical scenarios on a newborn mannequin, yielded poorer outcomes, with only one-third of the providers at endline able to correctly perform tactile stimulation and bag-mask ventilation. Not only were the scores relatively stagnant between years, performance actually declined between baseline and endline. The number of providers assessed was not large enough for statistical comparison.

### Observations of actual newborn resuscitation

During the course of the study, actual cases of newborn resuscitation were observed and evaluated as often as they presented during the observation visits. More resuscitations were seen in the endline assessment (40 cases (7.9%) out of 504 newborns observed) compared to the baseline assessment (18 cases (4.3%) out of 419 newborns observed). Although the numbers observed were too small for statistical comparison, it is encouraging to note that proportionally more resuscitations were observed in 2012. This could be attributable to the newborn resuscitation training and follow-up support occurring at these facilities as part of the intervention. Table 5 presents data from the resuscitations observed in 2012.

**Table 5:** **Observations of actual (non-simulated) newborn resuscitations of initially non-vigorous newborns, 2012**

**Newborn resuscitation step**	**n (N = 40)**
Suctions and/or rubs back for stimulation	32
Newborn not breathing after suctioning / stimulation	23
Ventilates correctly: first attempt	12
Ventilates correctly: second attempt	7
Ventilates correctly: third attempt	1
Ventilates at rate of 30–50 breaths/minute	9
Outcome of resuscitation: unsuccessful*	5
Outcome of resuscitation: successful	35

*3 of 5 of the unsuccessful resuscitations were noted to be fresh stillbirths.

In 17 of the 40 observed resuscitation cases (43%), the newborn began breathing in response to one or more of the initial resuscitation steps: drying, suction, and stimulation. When bag-mask ventilation was deemed necessary, it was performed correctly on the first attempt in less than half of the cases; only 45% (n = 9) of these newborns received ventilation at the correct rate of 30–50 breaths per minute.

Other noted gaps included effective communication with mothers, timely initiation of newborn resuscitation (within the first “golden minute”), and responding accurately to non-vigorous newborns. In the latter case, it is recommended that all non-macerated newborns should receive resuscitation attempts, even if there are no apparent signs of life initially, and the non-vigorous newborn should only be considered a stillbirth if there is no response to effective resuscitation.

Notes from the observations of actual resuscitations indicated some strengths in clinical care, as illustrated by the note of an observer, from a case in which the newborn survived:

*The mother was treated with respect, informed about the condition of the baby after [the] baby cried. The suctioning [was] done in front of the mother; no delays observed (2012)*

However, poor performance was also observed. Notes from another case in which the newborn did not survive highlight some of the gaps that contribute to bad outcomes for newborns and mothers:

*The mother was treated disrespectfully and not informed of the resuscitation of her baby. There was a delay of initiation of resuscitation, and the provider demonstrated inadequate knowledge; and there was no suction tube or penguin suction available. After the newborn death, the mother was counselled (2012)*

### Availability of supplies for newborn resuscitation and essential newborn care

**Figure 1:**
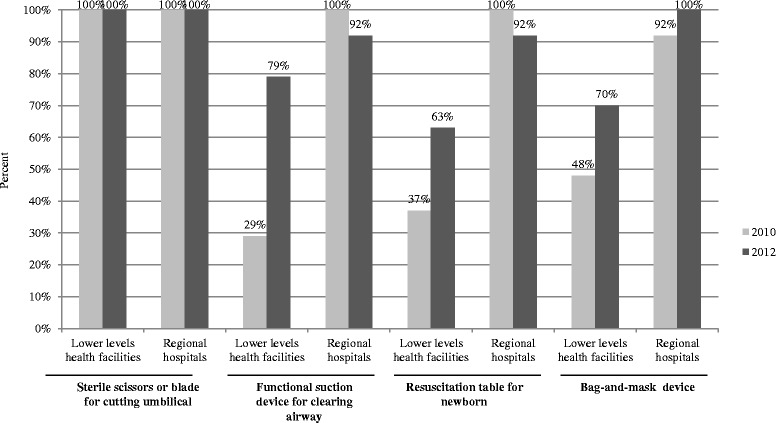
**Availability of key supplies and equipment for newborn care and resuscitation, by level of health facility, 2010 and 2012.**

Figure 1 shows an increase in the availability of selected equipment for newborn care and resuscitation. Lower-level health facilities were poorly equipped for newborn resuscitation at baseline and showed improvement by the end of the intervention, specifically, a 50% (*p* = 0.0001) increase in availability of simple suction equipment (i.e., Laerdal’s re-usable ‘penguin’ sucker). Regional hospitals had near-universal availability of resuscitation equipment at baseline, and this was sustained through follow up with the exception of one regional hospital, which had non-functional equipment at the time of the second assessment.

## Discussion

Results of the assessment following the intervention showed many significant improvements in the quality of newborn care, including skin-to-skin care, delayed cord clamping, breastfeeding within one hour of birth, and the overall index score for quality of newborn care. The results of health workers’ skills in newborn resuscitation, however, were disappointing; scores of providers conducting resuscitation scenarios using a newborn mannequin remained stagnant or even dropped at endline.

Some indicators, notably initiation of breastfeeding, showed a more pronounced improvement in lower-level health facilities than in regional hospitals, but the overall quality of care index score at endline was not significantly different between the levels. Breastfeeding of newborns within one hour is among the interventions with the greatest potential positive impact on child survival [1]. While an impressive increase of 32 percentage points in initiating breastfeeding was achieved among lower-level facilities, this critical, cost-effective intervention was still not universal (seen in 87% of deliveries) at endline.

According to the findings from the health worker knowledge and skills assessments, there were modest but significant increases in knowledge, but no increase in skills for newborn resuscitation using a mannequin. One possible explanation is that providers were unfamiliar with the skills assessment methodology (simulated resuscitation using a newborn mannequin). However, providers were supposed to be exposed to the mannequin during regular on-the-job practice and as part of the program’s strengthening of supportive supervision for quality assurance. It is quite possible that the findings reflect real, poor resuscitation skills of health care providers in maternity wards. Indeed, findings from Msemo et al. indicate that skills and practice of the steps of care related to the Helping Babies Breathe (HBB) program (stimulation, suction, and ventilation) were poorly performed at baseline in 9 facilities in Tanzania [15]. Other studies from low- and middle-income countries have shown similar barriers to newborn resuscitation, including limited provider knowledge and skills, inadequate training, and poor availability of equipment [25–28].

The low performance and lack of improvement in newborn resuscitation raises the question of what needs to be done to improve providers’ skills in newborn resuscitation, especially at lower-level health facilities with low delivery volume, where providers may not have the occasion to perform resuscitation frequently. To help address some of these deficiencies, the MOHSW and partners are currently implementing HBB and a clinical mentorship program throughout Tanzania to improve newborn resuscitation skills and provide essential equipment. These efforts include supportive supervision, refresher trainings, guided on-the-job practice, and SMS text reminders to providers.

According to the WHO, skin-to-skin contact for at least an hour immediately following birth and breastfeeding during this period is a high-impact intervention that supports the establishment of exclusive breastfeeding, assists with appropriate thermal care, and promotes bonding between mother and newborn [12]. While improvements in skin-to-skin care were notable – with the practice almost doubling overall – in the end, only 77% of newborns were being provided with this important, no-cost practice. A recent study of newborn care in Ethiopia also identified skin-to-skin care as a major gap in immediate newborn care [29].

Separating mothers and newborns, which was noted but not quantified in this study, is a particularly dangerous practice. Leaving newborns unobserved and unmonitored in the first critical few hours of life can significantly increase their risk for inappropriate thermoregulation and nutrition. Limiting maternal-newborn separation is particularly pertinent in light of critical shortages of health care providers at all levels of health facilities in Tanzania.

Although newborn resuscitation equipment was almost universally available at regional hospitals at both baseline and endline, this was not the case in the lower-level health facilities, where only one-third had the necessary equipment at baseline. Lower-level health facilities saw an increase in the availability of equipment – up to 70% – which is dramatic but not yet sufficient. The importance of having newborn resuscitation equipment cannot be overstated. The United Nations Commission on Life-Saving Commodities for Women and Children includes a bag-and-mask device for newborn resuscitation on its list of 13 affordable and effective but underutilized lifesaving commodities [30].

Recent findings by Ajaari et al. confirm that newborns delivered outside a health facility are more likely to die compared to newborns delivering in health facilities [31]. Findings from our current study confirm that improvements in facility-based newborn care can be achieved, but there are areas of persistent gaps, including newborn resuscitation and proper skin-to-skin care. As Tanzania continues to promote facility-based births with skilled attendants, investment in competent human resources and necessary infrastructure and supplies is urgently needed to ensure improved newborn survival. Training should continue to emphasize skin-to-skin care and initiation of breastfeeding within the first hour of life, with consideration for implementing UNICEF Baby-Friendly Hospital Initiative [32]. Meanwhile, the HBB program should help address gaps in training and equipment for newborn resuscitation during the Golden Minute after birth.

### Limitations

This evaluation of quality of care did not have a comparison arm, rather generated two point-in-time measurements. Despite this, we feel confident attributing changes in the health facilities in the study to the maternal and newborn quality improvement intervention, since there were no other documented improvement measures occurring at the same time. While other quality improvement initiatives, such as 5S, were in place by the MOHSW during the time of this program, none of these were actively underway in the MAISHA facilities during the time period under study, and we do not feel that other initiatives affected the results presented in the study. The study was conducted among regional hospitals, health centers, and dispensaries with at least one delivery per day taken from the annual delivery volume; our findings are not necessarily generalizable to other facilities. The Hawthorne or observer effect as a result of providers being observed may have introduced positive bias into the results of the observations. Nevertheless, this bias would presumably be similar during both the first and second round of data collection. Some of the observation steps were harder to observe than others. Notably, ‘initiate breastfeeding within one hour’ was difficult to observe since the observer did not stay continuously with the same woman for the whole hour after birth if there were other births occurring to observe. Further, the phrasing of the question was whether the provider *encouraged* the woman to initiate breastfeeding. This can be interpreted broadly and is not as informative as looking specifically at whether the woman was assisted with latching on or positioning the baby. Anecdotal feedback from observers indicated that the encouragement that the providers gave may not have been helpful enough to change mothers’ behavior in initiating breastfeeding if mothers were not inclined. In the calculation of index scores, missing data on newborn care were assumed to have a value of zero and included in the analysis. This could have resulted in an underestimation of quality in the newborn care index scores.

## Conclusion

Over the course of these two years, this newborn care quality improvement program was successful in raising the quality of essential newborn care services in the program facilities. Some gaps in newborn care were persistent, notably practical skills in newborn resuscitation. Current efforts to roll out HBB nationally, which involves training and equipment, including newborn mannequins for health care providers to practice resuscitation, may help in addressing these gaps. Continued investment in life-saving improvements to newborn care is a priority for reduction of newborn mortality in Tanzania.

## Abbreviations

BEmONC: Basic emergency obstetric and newborn care
HBB: Helping Babies Breathe
MDG: Millennium Development Goal
MOHSW: Ministry of Health and Social Welfare
USAID: United States Agency for International Development
WHO: World Health Organization
